# Supplementary material for: SARS-CoV-2 virus infection of Peromyscus leucopus demonstrates that infection tolerance is not limited to agents for which deermice are reservoirs
Source: bioRxiv. 2026 Mar 15:2026.03.13.711660. Preprint. [Version 1] doi: 10.64898/2026.03.13.711660 (PMC13060924; doi:10.64898/2026.03.13.711660)
Supplement: Supplementary file 1 [file media-1.docx]

**SARS-CoV-2 virus infection of** ***Peromyscus leucopus*** **demonstrates that infection tolerance is not limited to agents for which deermice are reservoirs**

Ana Milovic^1^, Johannes S. Gach^2^, Ioulia Chatzistamou^3^, Gema M. Olivarria^4^, Thomas E. Lane^4,5^, Donald N. Forthal^2,5^, and Alan G. Barbour^1,2^

1 Department of Microbiology & Molecular Genetics, School of Medicine, University of California, Irvine, Irvine, California, USA

2 Division of Infectious Diseases, Department of Medicine, School of Medicine, University of California, Irvine, California, USA

3 Department of Pathology, Microbiology & Immunology, School of Medicine, University of South Carolina, Columbia, South Carolina, USA

4 Department of Neurobiology & Behavior, School of Biological Sciences, University of California Irvine, Irvine, California USA

5 Department of Molecular Biology & Biochemistry, School of Biological Sciences, University of California Irvine, Irvine, California, USA

**Supplement**

Figure S1. In situ localization of SARS-CoV-2 RNA in lung tissue by RNAscope hybridization. Formalin-fixed, paraffin-embedded lung sections from *P. leucopus* or M. musculus and counter stained with hematoxylin were subjected to RNAscope in situ hybridization with a probe targeting SARS-CoV-2 genomic RNA. Red signal indicates the presence of viral RNA. The left panel is lung tissue of a *P. leucopus* animal inoculated with SARS-CoV-2 and at day 3 post-inoculation. It shows focal viral RNA localization within a peribronchial inflammatory infiltrate. The center panel is a lung section from K18-hACE2 transgenic M. musculus used as a positive control, demonstrating widespread alveolar infection. The right panel is of lung tissue from uninfected P. leucopus used as a negative control and showing absence of hybridization signal. 

Figure S2. Transcription of the Interferon Regulatory Factors Irf3 and Irf7 in the lungs of *P. leucopus* with (Virus) and without (Medium) infection by SARS-CoV-2 virus on day 3 or day 6 on infection. Panels A (Irf3) and B (Irf7) are box-whisker plots with means and 95% confidence intervals indicated. Panel C is a scatter plot with regression of transcripton of Irf7 on Irf3. Transcription is shown as natural logarithm of ratiio of either Irf3 or Irf7 reads to Gapdh reads. Linear regressions with coefficients of determination (*R*^*2*^) were determined separately for controls on days 3 and 6, virus-infected on day 3, and virus-infected on day 6.

Figure S3. Functional enrichment analysis of differentially expressed genes of whole brains of P. leucopus infected with SARS-CoV-2 virus. Gene ontology analysis was based on upregulated genes with a false discovery p value < 0.05 and a log2 fold change of ≥1.5 for day 3 (upper panel) or day 6 dpi (lower panel). The data for the analysis are provided in Table S5 The identification numbers for each of the listed terms are given in Table S3.
