# Supplementary material for: SARS-CoV-2 virus infection of Peromyscus leucopus demonstrates that infection tolerance is not limited to agents for which deermice are reservoirs
Source: bioRxiv. 2026 Mar 15:2026.03.13.711660. Preprint. [Version 1] doi: 10.64898/2026.03.13.711660 (PMC13060924; doi:10.64898/2026.03.13.711660)
Supplement: Supplementary file 2 [file media-2.docx]

SUPPLEMENTAL MATERIAL

Figure S1. In situ localization of SARS-CoV-2 RNA in lung tissue by RNAscope hybridization

Figure S2. Transcription of the Interferon Regulatory Factors Irf3 and Irf7 in the lungs of *P. leucopus* with (Virus) and without (Medium) infection by SARS-CoV-2 virus on day 3 or day 6 after infection. 

Figure S3. Functional enrichment analysis of differentially expressed genes of whole brains of *P. leucopus* infected with SARS-CoV-2 virus. 

Table S1. Differences in transcription in lungs between SARS-CoV-2-infected *P. leucopus* and controls on days 3 and 6 for 25,600 CDS. Abbreviations: CPM, count per million; FDR, false discovery rate

Table S2. Differences in transcription in lungs between SARS-CoV-2-infected *P. leucopus* and controls on days 3 and 6 for select differentially expressed genes by the criterion of fold change ≥1.0 up or down and a false-discovery rate *p* value <0.05. Abbreviations: FDR, false discovery rate

Table S3. GO terms associated with descriptions of processes and pathways

Table S4. . Targeted RNA-seq analyses of selected CDS in *P. leucopus* lungs per animal. Abbreviations: F, female; M, male.

Table S5. Targeted RNA-seq analyses of selected CDS in *P. leucopus* brain per animal presented as LN of the ratio of normalized reads to Gapdh reads. Abbreviations: V, treatment with virus; C, control; sex 0, female; sex 1, male
